# Detecting SNP markers discriminating horse breeds by deep learning

**DOI:** 10.1038/s41598-023-38601-z

**Published:** 2023-07-18

**Authors:** Siavash Manzoori, Amir Hossein Khaltabadi Farahani, Mohammad Hossein Moradi, Mehdi Kazemi-Bonchenari

**Affiliations:** Department of Animal Science, Faculty of Agriculture and Natural Resources, Arak University, Arak, Iran

**Keywords:** Bioinformatics, Animal breeding, Classification and taxonomy, Computational neuroscience

## Abstract

The assignment of an individual to the true population of origin using a low-panel of discriminant SNP markers is one of the most important applications of genomic data for practical use. The aim of this study was to evaluate the potential of different Artificial Neural Networks (ANNs) approaches consisting Deep Neural Networks (DNN), Garson and Olden methods for feature selection of informative SNP markers from high-throughput genotyping data, that would be able to trace the true breed of unknown samples. The total of 795 animals from 37 breeds, genotyped by using the Illumina SNP 50k Bead chip were used in the current study and principal component analysis (PCA), log-likelihood ratios (LLR) and Neighbor-Joining (NJ) were applied to assess the performance of different assignment methods. The results revealed that the DNN, Garson, and Olden methods are able to assign individuals to true populations with 4270, 4937, and 7999 SNP markers, respectively. The PCA was used to determine how the animals allocated to the groups using all genotyped markers available on 50k Bead chip and the subset of SNP markers identified with different methods. The results indicated that all SNP panels are able to assign individuals into their true breeds. The success percentage of genetic assignment for different methods assessed by different levels of LLR showed that the success rate of 70% in the analysis was obtained by three methods with the number of markers of 110, 208, and 178 tags for DNN, Garson, and Olden methods, respectively. Also the results showed that DNN performed better than other two approaches by achieving 93% accuracy at the most stringent threshold. Finally, the identified SNPs were successfully used in independent out-group breeds consisting 120 individuals from eight breeds and the results indicated that these markers are able to correctly allocate all unknown samples to true population of origin. Furthermore, the NJ tree of allele-sharing distances on the validation dataset showed that the DNN has a high potential for feature selection. In general, the results of this study indicated that the DNN technique represents an efficient strategy for selecting a reduced pool of highly discriminant markers for assigning individuals to the true population of origin.

## Introduction

DNA probes and sequences are two important indices in gaining a deep understanding of the evolution process, and the amount of DNA sequence data is rapidly increasing^1^. Single nucleotide polymorphism (SNP) is a new type of marker that includes many important characteristics for evaluating animals^2^, crops^3^, and human population structure^4^. At present, genomic data plays a critical role in a variety of biological contexts due to its numerous advantages. However, the curse of dimensionality (small n and large p) is a major limitation to their ability for practical applications. The lack of complete pedigrees and misidentification of parents affects the accuracy of genetic evaluations, and consequently, the efficiency of breeding programs. Identification of the discriminant SNP(s) process is one of the most appealing opportunities to exploit genomic data, for practical use, including determining the population of origin for unknown individuals^2^. Many researchers have widely investigated discriminant SNP(s) and genetic diversity^5–8^. Researchers can use such SNP markers for developing a cheap customized panel to trace the breeds. Furthermore, the SNP(s) can provide a reliable solution for the traceability of breed-specific branded products^9^.

In feature selection, researchers seek to identify key variables and eliminate annoying (or noisy) variables^10^. The same condition is true for biological data^11^, especially SNP markers. In various areas of breeding, we are always looking for SNP markers with enormous effects. Now, we import the issue to machine learning, especially the neural network approach. In genetics, this process is also known as Tag SNP Selection Problem (TSSP)^12^.

Mimicking the behavior of the biological brain in the nerve system is the base of Artificial Neural Networks (ANNs), which are the information processing tools^13^. Researchers have argued the shortcomings of ANN, including the complexity of analysis, computational cost, and time consumption. However, we must mention that ANN’s high prediction accuracy compensates its drawbacks to a great extent. Deep Neural Networks (DNN) have been employed to analyze biological data^14,15^. They have many applications in feature abstraction and selection^16,17^. DNNs were able to construct many biological prediction models^18^, but their power of feature selection had been ignored for individual discrimination.

The ANNs have recently been applied as a powerful statistical modeling technique for many areas of different biological data, especially in the animal sciences^19,20^. Fernández, et al.^21^ have indicated that ANNs were suitable to be used in fields of time series data for weekly milk prediction and clustering individuals in goat flocks. Ince and Sofu^22^ modeled data with ANN for the prediction of the sheep milk yield by using the back-propagation algorithm.

For feature selection (FS) based on ANN, a comparison was made in this study to discriminate among different horse breeds as well as to assign new individuals to their breed. Statistically, in the analysis of GWAS, all SNPs act separately and conduct the research with significant results. The consequence of this analysis obtains the identification of significant SNP markers, but the relationships between them are ignored. While the network approach is more reliable and logical monitoring all SNPs simultaneously leads to better results efficiently.

To obtain the best results, allele dosage has been applied to ANNs, which is a completely unbiased estimation. The Garson (weights) algorithm illustrates behavioral instability in the analysis, which can be considered a weakness^23^. Unlike most studies, Olden, et al.^24^ examined the performance of the Garson algorithm in the variable selection on simulated data, and have found that it has the lowest efficiency compared with other studied algorithms. Ibrahim^25^ showed that the Olden and Garson methods had the weakest results. The results of Fischer^26^ revealed that the Garson algorithm has a higher degree of stability in modeling non-linear relationships. Additionally, other studies have used the Garson and Olden algorithms, which are only applicable to ANN with a single hidden layer.

To the best of our knowledge, researchers had not investigated the potential of feature selection by ANN approaches for assigning individuals in horse breeds. We have analyzed the ANN’s potential to characterise, whether ANNs can be used as a tool for tackling the curse of dimensionality of SNP(s) data. We attempted to compare the DNN alongside a brief description of Garson and Olden methods to gain the relative importance of variables (SNP markers). While the DNN is a multiple hidden layer ANN, the two mentioned methods are compatible with a single hidden layer. This paper is one of the first studies to determine the discriminant SNP(s) on a large scale by using the sophisticated methods of ANN approaches. We have conducted this study intending to find distinct SNP markers to reduce the dimensions of the SNP panels as well as comparing different variable selection methods such as Garson and Olden through the ANN approach.

## Results and discussion

### Feature selection: comparison between three approaches

In the current research, we have used the three feature selection (FS) methods namely Olden, Garson, and DNN. Neural networks are commonly referred to as powerful and efficient statistical modeling techniques by various researchers^25^. Many studies have compared different FS methods^26–29^. The selection criteria for the variables in the DNN structure were the absolute value of the first hidden layer connection weights that they assumed as the regression coefficient. According to the DNN procedure, 4270 SNP markers had been selected for the rest of the analysis. The Garson and Olden algorithms led to a selection of 4937 and 7999 SNP markers for further analysis, respectively. The reason for choosing a more significant number of SNP tags for the Olden algorithm is the low transparency of the PCA plot. We must have mentioned that increasing the number of tags did not increase transparency anymore, this could be due to no linear relationship between SNPs number and PCA plot transparency. Moreover, the absolute increase of markers did not include a useful index for improvement unless the marker allele frequencies were different across subpopulations.

After the selection process of SNP markers, all SNP markers were sorted based on the calculated coefficient. The 460 top-rank SNP of each approach was selected, and all sub-SNP sets were compared to each other to find the common markers (Table 1). Table 1 represents the common SNP(s) in the prime 460 SNP markers. It indicates that all three methods had at least a 34% overlap (the average number of common SNPs is 158).

**Table 1:** Comparison among three feature selection methods based on prime 460 selected SNP markers.

	DNN	Garson	Olden
DNN	–	0.4309	0.5577
Garson	120	–	0.9810
Olden	167	185	–

The upper triangle represents the Spearman correlation among three methods for ranking markers. While the lower triangle represents the number of common tags between feature selection methods.

Regarding Table 1, we have found the lowest number of SNP markers between the DNN and Garson approaches. This phenomenon could be owing to the weights of the first layer in the two approaches. We have obtained the most significant number of SNP markers between Garson and Olden. This evidence shows that Garson and Olden had similar mechanisms for feature selection by using NN’s weights in the input-hidden and hidden-output layers. The Spearman correlation for coefficients of common markers indicated a strong relationship between Garson and Olden methods (98.10%). Also, the association obtained between DNN and Garson methods is 43.1%, which is confirmed by the number of common SNP markers.

In general, most of the studies have widely used the Olden and Garson approaches. The results of Olden, et al.^24^ revealed that the Olden method was the best overall methodology for processing and identifying the variable importance in the neural network, especially when the inputs had a weak or strong correlation with output. Fischer^26^ compared the Olden and Garson methods and reported that the results obtained by the Garson method are preferable and more stable than those obtained by the Olden method for nonlinear relationships. Findings from his study have shown that ranks obtained by the Garson approach may be more reliable than the Olden method, especially when those ranks are used for modeling nonlinear data such as positive and negative quadratics and interactive data. The results of these studies indicated that the Olden (Connection weights) method had an excellent performance for different assumptions and, Garson (Weights), as the ancestor of the weighted methods, had a various behavior in these studies.

All mentioned studies used the simulated or ecological data in which the maximum input variables were less than 20 variables. At first glance, both Olden and Garson’s algorithms used the input-hidden and hidden-output connection weights for calculating the importance of variables. The linear regression modeling habe been used as a control method on the real datasets for evaluating the input's significance in some studies^23,25^, and some others have used simulated data where the data have mostly contained the linear^24,28^ or semi-linear relationship^27^. However, the DNN approach could raise the performance and efficiency of the artificial neural network in circumstances where a large number of input variables (for example, genomic data of the globally equine breeds) have confronted the system.

### Feature selection: a comparison based on PCA analysis

**Figure 1:**
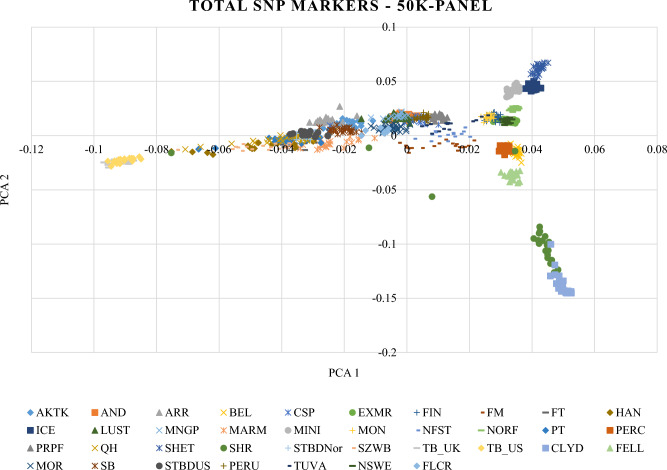
Animals were clustered based on principal components analysis (PCA) using all 50K SNP markers. PC1 and PC2 are shown on the X-axis and the Y-axis respectively. The horse breeds are demarcated using unique and different symbols and colors.

In the first place to assess the degree of divergence among samples, the principal component analysis (PCA) was applied to determine how the animals were allocated to the groups^30^. The actual coefficients of SNP markers have been obtained step by step according to the original PCA plot, which is according to the numerical analysis in mathematics. In other words, after choosing a new coefficient, the PCA plot was drawn, and the breed distinction was compared with the main PCA plot created by 50K SNP markers panel (Fig. 1). After marker selection and discovering the subsets of markers, PCA analysis was performed using all three sub-SNP(s) and total 50k SNP(s) available on SNP chip (Figures S1 (DNN), S2 (Garson), and S3 (Olden)).

The results indicated an excellent performance of PCA in distinct individuals into separated groups. PCA analysis has identified two subpopulations of Thoroughbred, (TB_UK & TB_US), as one breed, and a similar result was obtained for Standardbred (STBDNor & STBDUS) too. In Fig. 1, some breeds overlapped, but according to the symbols of each breed, we can say that these breeds are properly distinct from each other. Some breeds like Clyd, Shire, Shet, Ice, Mini, and TB (UK-US), were located in corners of the PCA plot, and this fact is due to the geographic boundaries of their countries (Table 5). In other words, these breeds belong to countries that have common borders. As a result, they might have more genetic resource exchanges with each other. Although STBD (including Nor and US) overlapped with Paint and Quarter breeds, they were completely separated by likelihood assessment. Asian breeds (AKTK, ARR, and CSP) were located near the center of the PCA plot and overlapped with Central European Breeds (CEB). It is highlighting this point that Asian breeds have a lot of common characteristics with CEB. The PCA analysis was performed for each method by selected SNP markers (Figs. S1 (DNN), S2 (Garson), and S3 (Olden)). The breed distinction is in good agreement with the main PCA plot created by 50K SNP markers (Fig. 1).

### Assessment of different methods and the number of SNP(s) to assignment

**Figure 2:**
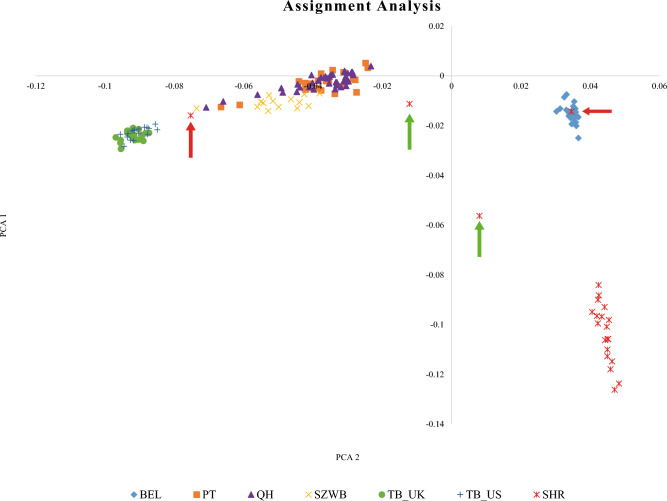
There are four individuals in the Shire breed, but two of them were recognized as hybrid animals (red arrows) by LLR analysis.

We have estimated the likelihood of assigning 795 individual genotypes to their known origins (or breeds) by the Paetkau, et al.^31^ approach. Although one particular breed (Shire) had at least one failure assignment by each method. In general, all three feature selection methods assigned most of the individuals to the right population. It resulted in a 9% reduction in the potential of the assignment procedure. Two individuals in the Shire breed failed in all subsets. Red arrows indicate these individuals in Fig. 2.

**Figure 3:**
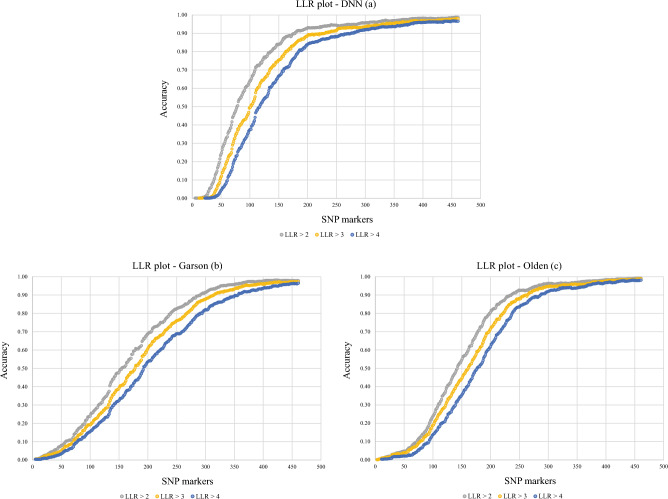
The success percentage of genetic assignment for DNN (**a**), Garson (**b**), and Olden (**c**) methods in the three stringency levels. The levels LLR > 2, 3 and 4 show that the individuals 100, 1000 and 10000 times are more likely to be assigned to the right population than the other one. The success rate of 70% in the analysis was obtained by three methods with the number of markers of 110, 208, and 178 tags for DNN, Garson, and Olden methods, respectively.

With the analysis of assignment and concerning values of LLR, obtained results showed that one failure was recognized as Belgian breed by three methods, and the other one was known as different breeds like Paint, Quarter, Swiss warmblood, and Thoroughbred-US. By using three methods, the first individual has 97.30% accuracy to be assigned to the correct race (Shire). By DNN, and Olden approaches, the second individual also had 91.89% accuracy for being appointed into the right breed. For further explanation, these failures might be due to hybrid or crossbreeding parentage. There were two Shire individuals in the center of the PCA plot (Fig. 2); the assignment method indicated that they belong to their breed (Green arrows). In Fig. 3, we have demonstrated the correctness plots for three feature selection algorithms at various strict levels.

As shown in Fig. 3, all three methods revealed different behavior for the success percentage of genetic assignment. In the DNN, the success rate in selecting the correct animal breed was more than in the other methods. The sufficient number of SNP markers required to correctly assign an unknown animal to its exact breed/origin at different threshold levels (90%, 95%, and 98%) have been shown for DNN, Garson, and Olden methods in Table 2.

**Table 2:** The number of markers required in each method for assigning an animal to its breed at different threshold levels of the LLR analysis.

Log(10)	90%			95%			98%		
	DNN	Garson	Olden	DNN	Garson	Olden	DNN	Garson	Olden
1	144	264	205	202	298	242	348	358	363
2	179	291	236	287	327	293	386	419	394
3	230	315	264	330	368	342	*	*	*
4	276	357	287	378	427	380	*	*	*

To calculate these numbers, the sophisticated analysis of LLR was performed individually with a certain number of markers. Using 460 top-rank SNP markers selected by each method, in each step of the LLR analysis, one SNP marker was added to the rest of the markers used in the earlier step.

*It requires more than 460 markers.

We have accurately calculated the percentages of individuals and correct assignments for different numbers of SNP markers. Testing the performance of each approach has been done at four different levels of LLR analysis. We found that DNN performed better than the other two approaches by achieving 93% accuracy at the most stringent threshold (LLR > 4) (Table 3). In this section, the Garson method did not perform well.

**Table 3:** Correct assignment of an individual by three Methods.

# SNP	LLR > 1			LLR > 2			LLR > 3			LLR > 4		
	DNN	Olden	Garson	DNN	Olden	Garson	DNN	Olden	Garson	DNN	Olden	Garson
50	353 (44.4)	50 (6.29)	100 (12.58)	191 (24.03)	38 (4.78)	64 (8.05)	93 (11.7)	31 (3.9)	39 (4.91)	35 (4.4)	17 (2.14)	27 (3.4)
75	524 (65.91)	130 (16.35)	177 (22.26)	377 (47.42)	92 (11.57)	119 (14.97)	272 (34.21)	73 (9.18)	93 (11.7)	171 (21.51)	44 (5.53)	74 (9.31)
100	624 (78.49)	274 (34.47)	256 (32.2)	510 (64.15)	190 (23.9)	190 (23.9)	395 (49.69)	151 (18.99)	152 (19.12)	298 (37.48)	110 (13.84)	123 (15.47)
125	681 (85.66)	451 (56.73)	358 (45.03)	613 (77.11)	319 (40.13)	269 (33.84)	518 (65.16)	256 (32.2)	219 (27.55)	433 (54.47)	187 (23.52)	181 (22.77)
150	722 (90.82)	550 (69.18)	483 (60.75)	668 (84.03)	434 (54.59)	381 (47.92)	600 (75.47)	354 (44.53)	311 (39.12)	531 (66.79)	282 (35.47)	258 (32.45)
200	755 (94.97)	705 (88.68)	629 (79.12)	740 (93.08)	641 (80.63)	546 (68.68)	706 (88.81)	568 (71.45)	483 (60.75)	668 (84.03)	492 (61.89)	426 (53.58)
250	760 (95.6)	761(95.72)	698 (87.8)	751 (94.47)	735 (92.45)	657 (82.64)	727 (91.45)	699 (87.92)	604 (75.97)	701 (88.18)	663 (83.4)	547 (68.81)
300	774 (97.36)	774 (97.36)	759 (95.47)	762 (95.85)	766 (96.35)	727 (91.45)	745 (93.71)	752 (94.59)	697 (87.67)	732 (92.08)	731 (91.95)	653 (82.14)
350	781 (98.24)	776 (97.61)	776 (97.61)	771 (96.98)	770 (96.86)	760 (95.6)	759 (95.47)	761 (95.72)	742 (93.33)	745 (93.71)	746 (93.84)	712 (89.56)

Number of individuals assigned correctly (percentage).

The results revealed that the DNN outperformed other methods with fewer SNP markers. Generally, about 500 discriminant SNP markers enabled us to assign new individuals to the right groups using different ways. There are some issues related to the comparison of results in this study with other ones. First, many previous studies used another type of marker with only a limited number of tags^32–35^. Second, there were different methods in several studies^36^. Maudet, et al.^32^ found out that, by using 23 microsatellite loci, they could be assigned more than 90% of individuals to their breed. Negrini, et al.^37^ used the limited set of available SNP markers for an individual assignment. Aiming to determine the range of the minimum number of SNP markers (from 60 to 140), Wilkinson, et al.^38^ worked for assigning individuals in 17 Bovine breeds.

## Model validation

### PCA and LLR analysis for validation data

**Figure 4:**
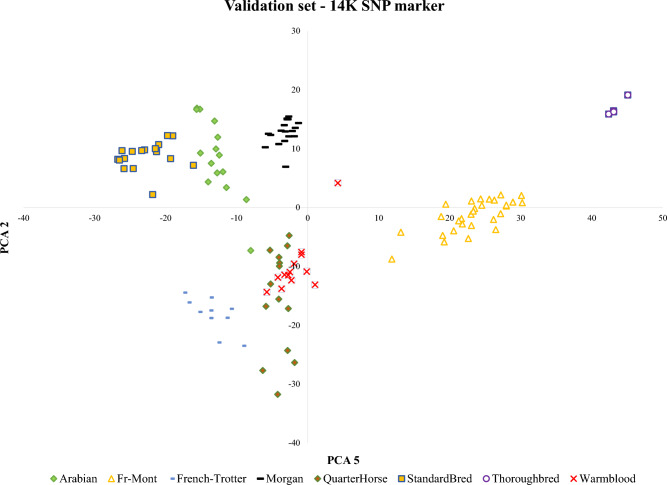
PCA analysis of all common SNP markers between training and validation data (14K SNP markers)

We have used a separate dataset to test the model. Firstly, we have applied the PCA analysis to find the relationship among the breeds like the training dataset (Fig. 4).

In Fig. 4, the Quarter and Warmblood have a small overlap. We identified and extracted the selective SNP markers of 3 feature selection methods (from panel 50K) in the evaluation dataset. Common extracted SNP markers were maintained for later analysis. We have isolated and extracted 839 (Fig. S4—DNN) from 4270 for DNN, 370 (Fig. S5—Garson) from 4940 for Garson, and 1718 (Fig. S6—Olden) from 7999 for Olden approaches in the validation data-set, respectively. Then, we have found the 85 (DNN), 15 (Garson), and 49 (Olden) SNP markers in the evaluation set based on the 460 top-rank SNP markers in the training set, respectively. The LLR analysis was performed for two series of data extracted from the test data and the results have been presented in Table 4.

**Table 4:** The selected SNP markers in the training dataset for each method and the number of common SNP markers were identified in the validation dataset.

	Train set (50K SNP markers)			Test set (14K SNP markers)		
	DNN	Garson	Olden	DNN	Garson	Olden
Total selected SNP(s)	4270 (99.74)	4940 (99.87)	7999 (99.74)	839 (100)	370 (100)	1718 (100)
First Top rank 460 SNP(s)	460 (98.86)	460 (99.62)	460 (99.62)	85 (97.5)	15 (60)	49 (94.17)

We have presented the estimated accuracy for each dataset in the parentheses.

The results of this section revealed that all three artificial neural networks had an excellent performance. The Garson method with a minimum number of markers (fifteen) had a 60% accuracy, which may be due to the low number of animals and the distinction between the source in the test data, because there are significant differences between the countries of Switzerland, France, and England (the continent of Europe) and the countries of the Middle East and the Americas (Asian and American continents).

By using one dataset, there is a possibility to observe a negligible amount of kinship relationships. Because all individuals are sampled from one herd, kinship relationships are practically inevitable in the research. Therefore, using new data from other sources reduces the probability of kinship among individuals. If unknown or novel information is introduced to the desired network, the least errors will get. Previously obtained results of the network were reliable enough for DNN to infer the right class of novel information precisely. In this case (DNN), the system undoubtedly possesses much power and much success in correctly determining the essential features.

### Neighbor-Joining tree of allele-sharing distances for validation data

For a better understanding, we have used the Neighbor-Joining tree of allele-sharing distances on the validation dataset. Neighbor-Joining analysis performs better than PCA analysis on topics such as breed-level differentiation, the intermingling of breeds, outliers, genetic isolation, etc. First, we have analyzed whole genomic data (32419 SNP markers, 120 horses) to show the breed-level differentiation in validation data (Fig. S7).

Then, the Neighbor-Joining analysis was done for each obtained dataset (Fig. S8 (DNN), S9 (Garson), S10 (Olden)) to demonstrate the breed distinction in comparison to the whole data. In Fig. S7, except for two groups (Quarter Horse and Warmblood) and despite the low amount of SNP markers, the rest of the breeds were in their real groups. It is critical to consider that two breeds (Quarter Horse and Warmblood), may have an unusual overlap due to the low number of markers.

We have drawn Fig. S8 by using the markers selected by the DNN. It is noteworthy that the classification of individuals is mostly successful, and there is no significant overlap between breeds. The Neighbor-Joining plot (Fig. S9) drawn by the selected markers of the Garson method did not have a good quality in terms of the classification of individuals. In Fig. S9, there was a great deal of unusual overlap between the breeds, and only the Thoroughbred was identified as a pure breed due to the small number of individuals. The number of outsiders in the results of this dataset was very high (red arrows).

The Olden method had the same performance similar to the DNN and whole data (Fig. S10). In a way, its plot was promising. Perhaps the only disadvantage of the Olden method compared to the other two is that despite the high number of SNP markers, two individuals (Arabian-3 and QuarterHorse-1) still have been identified as outsiders.

## Conclusion

We have used the weights of the first hidden layer of the DNN, for selecting and ranking variables (SNPs). Artificial neural networks (ANNs) will receive a great deal of attention in the various scientific fields, given that they are powerful statistical modeling techniques. However, in an attempt to provide useful insights into the contributions of the input (independent) variables in the prediction process, they have been labeled as the “black box” technique. As mentioned earlier, many published studies had been conducted to clarify the interpretation of the connection between the neurons in ANN.

By comparing the results, the Garson and Olden procedures only work with a single hidden layer and single output unit, while multiple layer networks (DNN) do not suffer these limitations. Regarding log-likelihood ratio (LLR) for the individual assignment, the obtained results by this research revealed that ANN’s feature selection methods could be used for genomic data, especially for dimension reduction by DNNs. This finding solves the most critical issue for genetics researchers in dealing with the considerable dimension of data. Researchers can use DNN in the field of animal sciences because of the high performance of breed discriminants. Researchers in the field of genetics and breeding are seeking to reduce the number of biomarkers to find a link between the observed phenotype and these markers.

The result of this study showed that the DNN has a high potential for feature selection in genomic data along with more flexibility in the application of ANNs in the field of animal sciences. Results also showed that using the connection weight of the first hidden layer in a DN Network provides the possibility to reach a high optimum level of accuracy for ranking and selecting the variables (SNP(s)). Another conclusion of this research is that the most critical weights for output values of every variable in a DN Network are the weights in the first hidden layer because all connected loads of the next layers are functions of the first layer's connected load. If three analyzes of PCA, LLR, and Neighbor-Joining achieve the desirable results, we will get the real discriminative features.

It is necessary to point out that the results of this study shed some lights on the using of DN Networks (especially pattern recognition) in genetics and breeding. Feature selection in the genetic field particularly on SNP markers is in the infancy period. The computation time will be reduced significantly. It should also be noted that the DNN network is increasing computing time but it was decreasing the error rate significantly. It can open a new opportunity to extend human insights.

Finally, we think that this will be a fruitful approach to the study of existing domestic populations, such as inferior local breeds and strains in developing countries. In general, the present paper highlighted the importance of variable selection from the varying point of view, including the socio-economic perspective (for developing a low-cost customized assay for assigning the breeds or tracing the origin of animal products derived from diverse species).

## Materials and methods

### The data for training ANN

A total of 795 animals from 37 breeds of horse populations were genotyped by using the Illumina SNP 50k Bead chip (Illumina, San Diego, CA, USA). Petersen et al.^7^ have already described the comprehensive description and necessary details of data mining. In summary, Table 5 has given the breed names, the ID of breeds, the geographic origin, minor allele frequency (MAF), Heterozygosity, and the number of animals. Genotype data are coded as the number of reference SNP allele carries, that is, 0 (for AA), 1 (for AB), and 2 (for BB). In the present study, a further filtration for the call rate (the proportion of SNP genotypes) less than 99% was used to discard the missing genotypes^39,40^.

**Table 5:** The name, identification code, geographic origin, size of samples (N), minor allele frequency (MAF), and observed heterozygosity (HO) of different horse breeds (Training dataset).

Breeds	ID	Origin	N	MAF	H_O_	Breeds	ID	Origin	N	MAF	H_O_
Akhal-Teke	AKTK	Turkmenistan	19	0.235	0.360	Morgan	MOR	United States	40	0.226	0.350
Andalusian	AND	Spain	18	0.229	0.353	New Forest Pont	NFST	England	15	0.217	0.340
Arabian	ARR	Middle East	24	0.240	0.365	North Swedish Horse	NSWE	Sweden	19	0.210	0.332
Belgian	BEL	Belgium	30	0.209	0.330	Norwegian Fjord	NORF	Norway	21	0.209	0.330
Caspian	CSP	Persia	18	0.223	0.347	Paint	PT	United States	25	0.244	0.369
Clydesdale	CLYD	Scotland	24	0.205	0.326	Percheron	PERC	France	23	0.209	0.330
Exmoor	EXMR	Great Britain	24	0.210	0.331	Peruvian Paso	PERU	Peru	21	0.222	0.345
Fell pony	FELL	England	21	0.212	0.334	Puerto Rican Paso Fino	PRPF	Puerto Rico	20	0.219	0.342
Finnhorse	FIN	Finland	27	0.209	0.331	Quarter Horse	QH	United States	40	0.245	0.369
Florida Cracker	FLCR	United States	7	0.229	0.353	Saddlebred	SB	United States	25	0.233	0.358
Franches-Montagnes	FM	Switzerland	19	0.221	0.344	Shetland	SHET	Scotland	27	0.208	0.329
French trotter	FT	France	17	0.242	0.367	Shire	SHR	England	23	0.211	0.333
Hanoverian	HAN	Germany	15	0.252	0.377	Standardbred—Norway	STBDNor	United States	25	0.238	0.363
Icelandic	ICE	Iceland	25	0.212	0.334	Standardbred—US	STBDUS	United States	15	0.241	0.366
Lusitano	LUST	Portugal	24	0.228	0.351	Swiss Warmblood	SZWB	Switzerland	14	0.252	0.377
Mangalarga paulista	MNGP	Brazil	15	0.224	0.348	Thoroughbred—UK/Ire	TB_UK	England	19	0.275	0.399
Maremmano	MARM	Italy	24	0.239	0.364	Thoroughbred—US	TB_US	England	17	0.275	0.399
Miniature	MINI	United	21	0.210	0.331	Tuva	TUVA	Siberia	15	0.216	0.339
Mongolian	MON	Mongolia	19	0.213	0.335	–	–	–	–	–	–

The MAF statistic shows the average minor allele frequency for the SNP(s) after data mining. The mean value of MAF over all samples was estimated 0.226704 and the minimum and maximum of MAF were observed in Clydesdale and Thoroughbred-UK/Ire breeds respectively (0.2047 & 0.2748).

Moreover, raw predictor variable data (SNP matrix) is used as the input variable in ANN. It is assumed that each of these markers represents a mathematical variable that can only hold 3 inputs (0, 1, and 2).

### The data for testing and validation methods

To assess the performance of the ANN methods, learning and evaluation were performed using two separate datasets, respectively. The testing dataset contains 120 individuals from eight breeds (Table 6 includes the sample information). You can find all the details and information about the validation data in the article by Schaefer, et al.^41^. Data preprocessing included extracting common SNP markers between panels of 50K and 2M. This process resulted in the identification of 32K markers, and 14K of these markers remained after quality control (call rate 99%) for further analysis.

**Table 6:** The name, identification code, geographic origin, size of samples (N), minor allele frequency (MAF), and observed heterozygosity (HO) of different horse breeds (Validation dataset).

	Breed	ID	Origins	Continents	N	Percent	MAF	H_O_
1	Arabian	ARR	Middle East	Asia	15	12.50	0.2419	0.3667
2	Fr-Mont	FM	Switzerland	Europe	29	24.17	0.2195	0.3426
3	French-Trotter	FT	France	Europe	10	8.33	0.2371	0.3618
4	Morgan	MOR	United States	America	18	15.00	0.2268	0.3507
5	Quarter horse	QH	United States	America	14	11.67	0.2477	0.3727
6	Standard breed	STBD	United States	America	17	14.17	0.2503	0.3753
7	Thoroughbred	TB	England	Europe	4	3.33	0.3758	0.4692
8	Warmblood	SZWB	Switzerland	Europe	13	10.83	0.2477	0.3727
	Total				120	100	–	–

The mean value of MAF statistic over all samples was estimated 0.2585 and the minimum and maximum of MAF were observed in Franches Montagnes and Thoroughbred breeds, respectively (0.2195 and 0.3758).

### ANN model and construction

**Figure 5:**
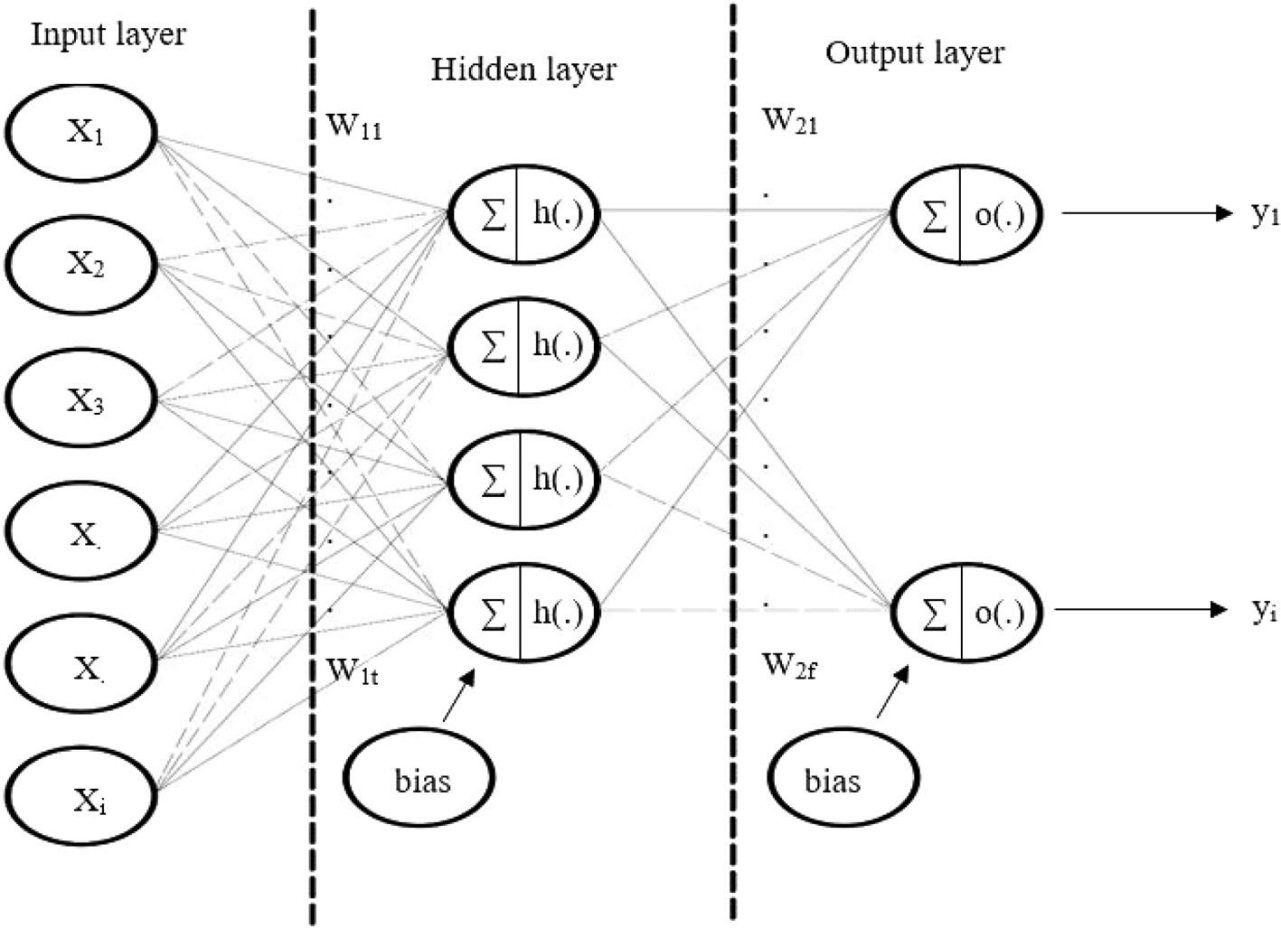
Single hidden layer network structure that has been used in most studies. Most of the elements of the network, like the particular bias for each neuron, are ignored in this figure for better understanding.

Artificial neural networks represent complex structures that are generated by fundamental units (elements) called neurons^22^. Neurons and their connections create a specific network architecture such as multilayer perceptron (MLP), self-organizing map (SOM), etc.^13^. In terms of genomic data analysis, we used two types of ANN architecture. The first one is a feed-forward multilayer perceptron (DNN) with two hidden layers, and the second one is a standard single hidden layer (ANN) with a back-propagation algorithm for the weight adjustments^42,43^. In Figure 5, The architecture of a single hidden layer ANN has been shown for better understanding. Neural net^44^ and Neural Net Tools^45^ packages were applied by R software (version 3.4.0)^46^ to select informative and unique SNP markers that are within each breed. The mentioned algorithms (Garson and Olden) have been utilized by ANN to detect the relative importance of variables for the breed diversity characterization.

The large dimension of the SNP-panel leads to a stack overflow error in the computing process. De Oña and Garrido^29^ have proposed the usage of a set of neural networks instead of a single one. In contrast to^29^ in the present work, the high-density SNP chip was partitioned into the sub-datasets with the same dimension and were used as input to identify the discriminant SNP(s).

### Feature selection: Garson and Olden

Weights (Garson approach), had been described by Garson^47^ and has also been modified by Goh^48^. It was used to identify the relative importance of input variables by the calculated weights within connections in a supervised neural network. The Garson approach indicates relative importance values as the absolute magnitude ranging from zero to one (0-1). Olden and Jackson^49^ had proposed connection weights, also known as the Olden approach that has been used in this research.

### Feature selection: DNN approach and its architecture

For the DNN approach, the ANN with two hidden layers was used to identify the discriminant SNP(s) within breeds. Many combinations exist for selecting the number of nodes in the hidden layer^50^. The optimal number of nodes in the first and second hidden layers detected 40 and 38 nodes after testing a range of combinations. Finally, ANN with Garson and Olden algorithms contained 40 nodes in the hidden layer.

We have used the final fitted weights of the neural network for selecting the genetic markers. In the DNN approach, we assumed there was a linear relationship between the variable and the response^12^. We considered the SNP markers to retain a direct relationship with the horse breeds. (Eq. 1).

1 $${\rm Y}={\rm Xg}+{ \rm e}$$

where *Y* is the matrix of observed values for the desired breeds, *g* is a vector of weights of SNP markers, and *e* is the vector of residual terms. *X* is known as the design matrix that relates the elements of *g* to its corresponding element in *Y*. Assuming that higher coefficient values in this (regression) equation have a significant effect on the output variable, the absolute maximum weight obtained by DNN led to the selection of SNP markers that caused the diversity of the breeds.


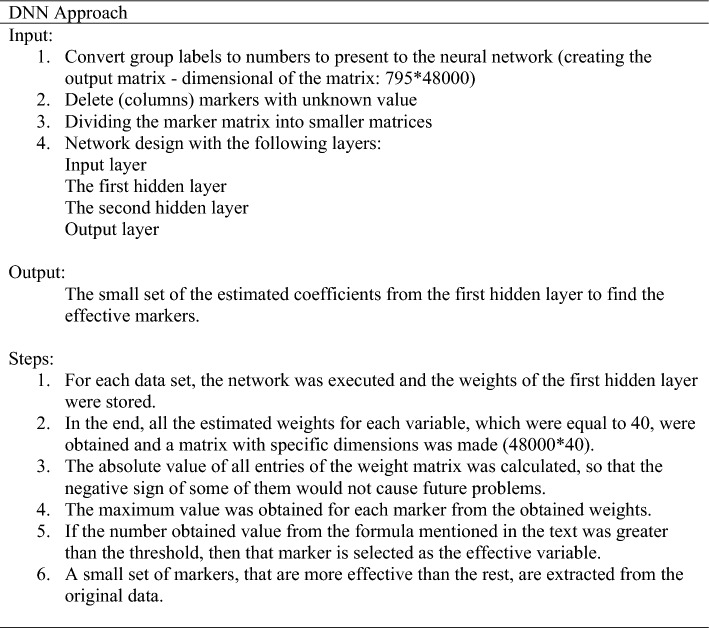


**Figure 6:**
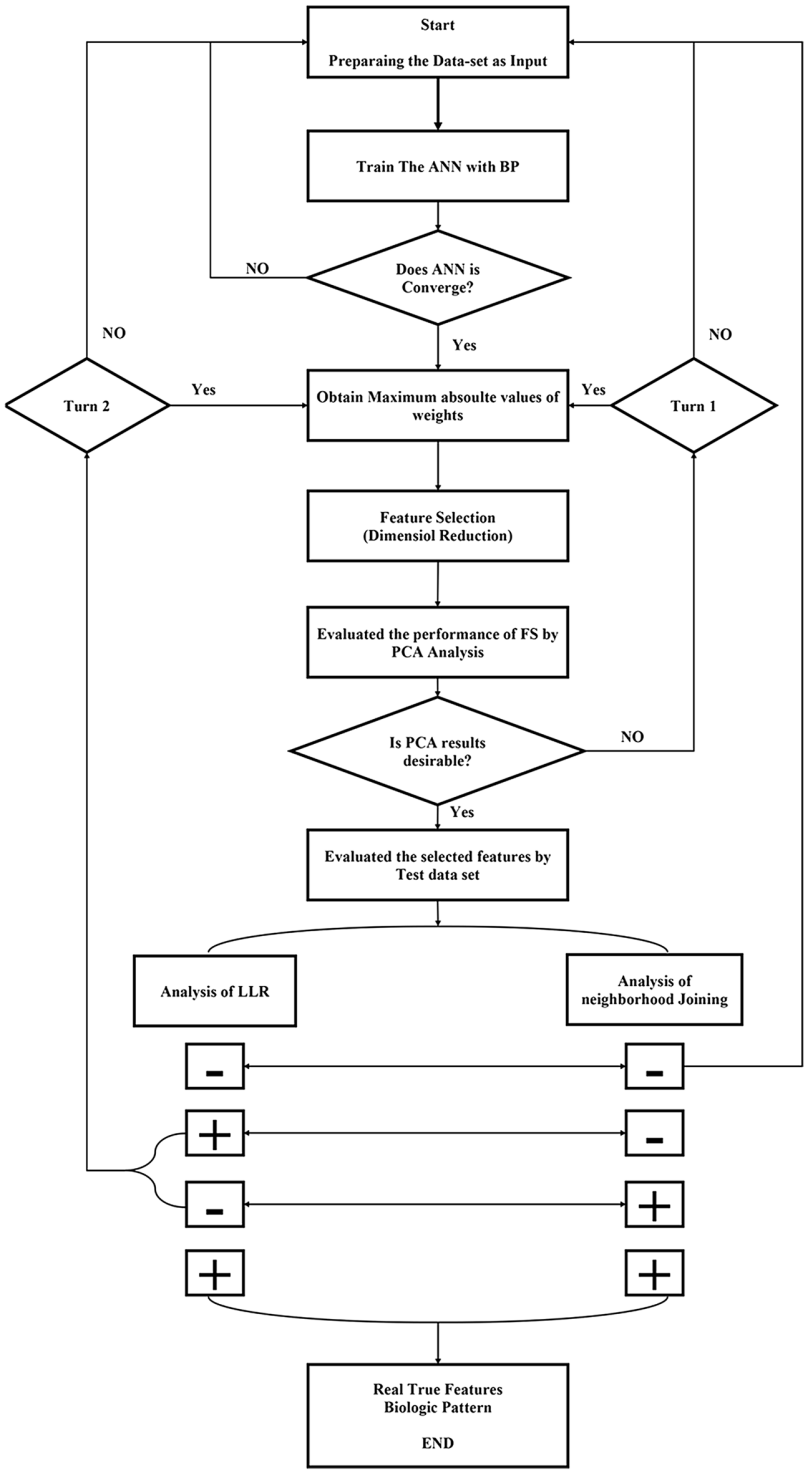
Flowchart of research used in the present study

Figure 6 shows the whole analysis process. The researchers must determine the features according to Eq. (2), after the convergence of the neural network (Fig. 6). Feature selection is based on the absolute value of the weights of the first hidden layer. It should be noted that 40 weights have been calculated for each variable. In this step, the maximum value is obtained for each variable. If the obtained value was greater than the coefficient of Eq. (2), then that variable was selected as the effective SNP marker.

2 $$E\left(\left|{W}_{ij}\right|\right)+\sqrt{Var\left(\left|{W}_{ij}\right|\right)}$$

By considering Eq. (2), it is assumed that all variables are doing their job with maximum potential. Then, a selection threshold was defined to choose a small set of variables. As previously described, in this status, the effects of all variables are not estimated equally and we see the minimum and maximum values among them. The reason for assuming maximum potential is that we do not know what is the actual effect of each variable in biological data. Therefore, we considered every marker on the same level and allowed them to make their inferences and results. Regarding stages Turn 1 and Turn 2, it can be explained that sometimes the result of feature selection in subsequent analyzes is not desirable. Finally, further analysis to evaluate the individual assignment accuracy and qualify all three sub-SNP sets was done by a manual script in R software version (3.4.0).

### Individual assignment analysis

There are several available approaches for genetic assignment^31,51,52^. The method of Paetkau, et al.^31^ has been used for the assignment analysis (as had been described by^38^), and it had high effectiveness on individual assignment when high levels of genetic differentiation between reference populations existed^52^. It is noteworthy that the SNP markers were applied instead of the microsatellites. We have calculated the log-likelihood ratios (LLR) to accurately assess the performance of the assignment procedure. The log-likelihood ratios (LLR) will be calculated by comparing the probability of an individual assigned to its real population to the probability of it assigned to another population (Eqs. 3 and 4).

3 $$LLR={log}_{10}\left(T\left(\mathrm{g}|{i}_{a}\right)\right)-{log}_{10}\left(T\left(\mathrm{g}|{i}_{b}\right)\right)$$

where,

4 $${log}_{10}\left(T\left(\mathrm{g}|i\right)\right)=\sum_{j}{log}_{10}\left(T\left({g}_{jk{k}{^\prime}}|i\right)\right)$$

Different stringency thresholds are applied as confidence levels of assignment precision. Four stringency levels were used: LLR > 1, 2, 3 & 4, which means a multi-locus genotype should be 10, 100, 1000 & 10000 times more similar to the true population rather than the other one. If a calculated LLR value was lower than the selected stringency levels, the individual genotype would fail to assign to its unique origin. In other words, it would assign to the pseudo reference population. The correct assignment of an individual genotype to its known origin occurred when the calculated LLR was greater than the selected stringency levels.

The aim of evaluating a classification model is to evaluate and understand its flexibility, behavior, and prediction ability in dealing with new or unknown samples.

### Ethics statement

Training Data-set: DNA sampling was limited to the collection of blood by jugular venipuncture performed by a licensed veterinarian or from hairs pulled from the mane or tail by the horse owner or researcher. All animal work was conducted in accordance with and approval from the international and national governing bodies at the institutions in which samples were collected (the University of Minnesota Institutional Animal Care and Use Committee (IACUC); the University of Kentucky IACUC; the University College Dublin, Animal Research Ethics Committee; Swiss Law on Animal Protection and Welfare; the Ethical Board of the University of Helsinki; the Animal Health Trust Clinical Research Ethics Committee; Norwegian Animal Research Authority; UK Home Office License; and the Lower Saxon state veterinary office).

Testing Data-set: DNA samples were previously collected with approval from the Animal Care and Use Committees at the respective institutions. All animal work was performed in accordance and with approval from international and national governing bodies at the institutions where the samples were collected (University of Minnesota Institutional Animal Care and Use Committee (IACUC); University of California, Davis Institutional Animal Care and Use Committee (protocol #17491); University of Kentucky Institutional Animal Care and Use Committee (IACUC); Ethics Committee for Animal Experiments in Uppsala, Sweden (Number C121/14); Institutional animal care and use committee at Cornell University (protocol 2008-0121); University of California, Davis IACUC 19205; Hebrew University’s approval number AG-23476-07; Institutional Animal Care and Use Committee (IACUC), the Lower Saxony state veterinary office- registration number 11A 160/7221.3-2.1-015/11, 8.84-02.05.20.12.066; University of Sydney Animal Ethics Committee: AEC APPROVAL NUMBER: N00/9-2009/3/5109; permit no. BE75/16, veterinary service of the Canton of Bern; Institutional ethics committee of the University of Veterinary Medicine Vienna Good Scientific Practice guidelines and national legislation; Italian Ministry of Agricultural, Food and Forestry Policies (Mipaaf); Ethical Committee of the Canton of Bern (BE33/07, BE58/10 and BE10/13)) No commercial animals were used in this study. Written informed client consent describing the purpose and duration of the study, procedures, potential risks and benefits and containing study contact information were obtained from private owners.

## Supplementary Information

Supplementary Information.

## Data Availability

Training Data-set: All SNP genotype data are available at the NAGPR Community Data Repository (animalgenome.org) for the purpose of reconstructing the analyses. The only exception is the data collected from the Tennessee Walking Horse, which, under agreement from the granting agency (to the University of Minnesota from the Foundation for the Advancement of the Tennessee Walking Show Horse (FAST) and the Tennessee Walking Horse Foundation (TWHF)), is only available under a Material Transfer Agreement (MTA) between interested individuals and the University of Minnesota. Testing Data-set: Whole genome sequences are available in the following NCBI BioProjects: PRJEB14779, PRJNA273402, and PRJEB10098. Additional sequences are restricted in availability due to pre-existing material transfer agreements and can be requested by contacting the contributing investigator in Additional file 1: Table S1. Genotypes for horses on the MNec2M array will be released upon publication. Genome positions for all 23 million discovered SNPs have been submitted to dbSNP as well as the European Variation Archive.
