# Supplementary material for: Detecting SNP markers discriminating horse breeds by deep learning
Source: Sci Rep. 2023 Jul 18;13:11592. doi: 10.1038/s41598-023-38601-z (PMC10354035; doi:10.1038/s41598-023-38601-z)
Supplement: Supplementary file 1 — Supplementary Information. [file 41598_2023_38601_MOESM1_ESM.docx]

**Detecting SNP markers discriminating horse breeds by Deep learning**

**Running title:** Feature selection in horse genome

**PCA plot of each method – Training data**


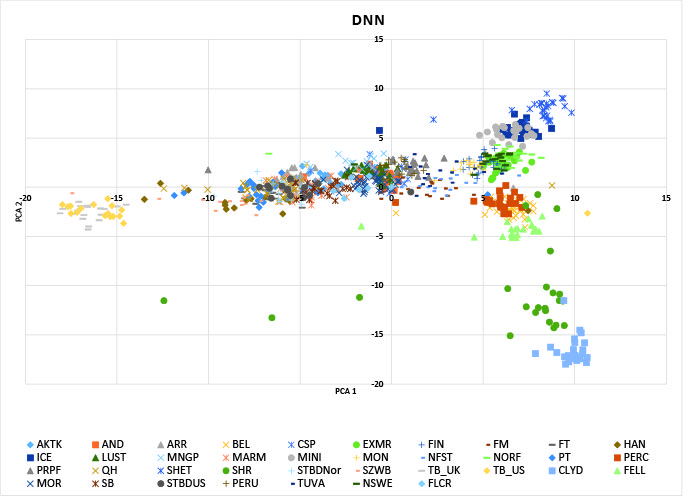


Figure S1 Animals were clustered based on principal components analysis (PCA) using 4270 SNP markers (DNN). PC1 and PC2 are shown on the X-axis the Y-axis respectively. The horse breeds demarcated using unique and different symbols and colors.


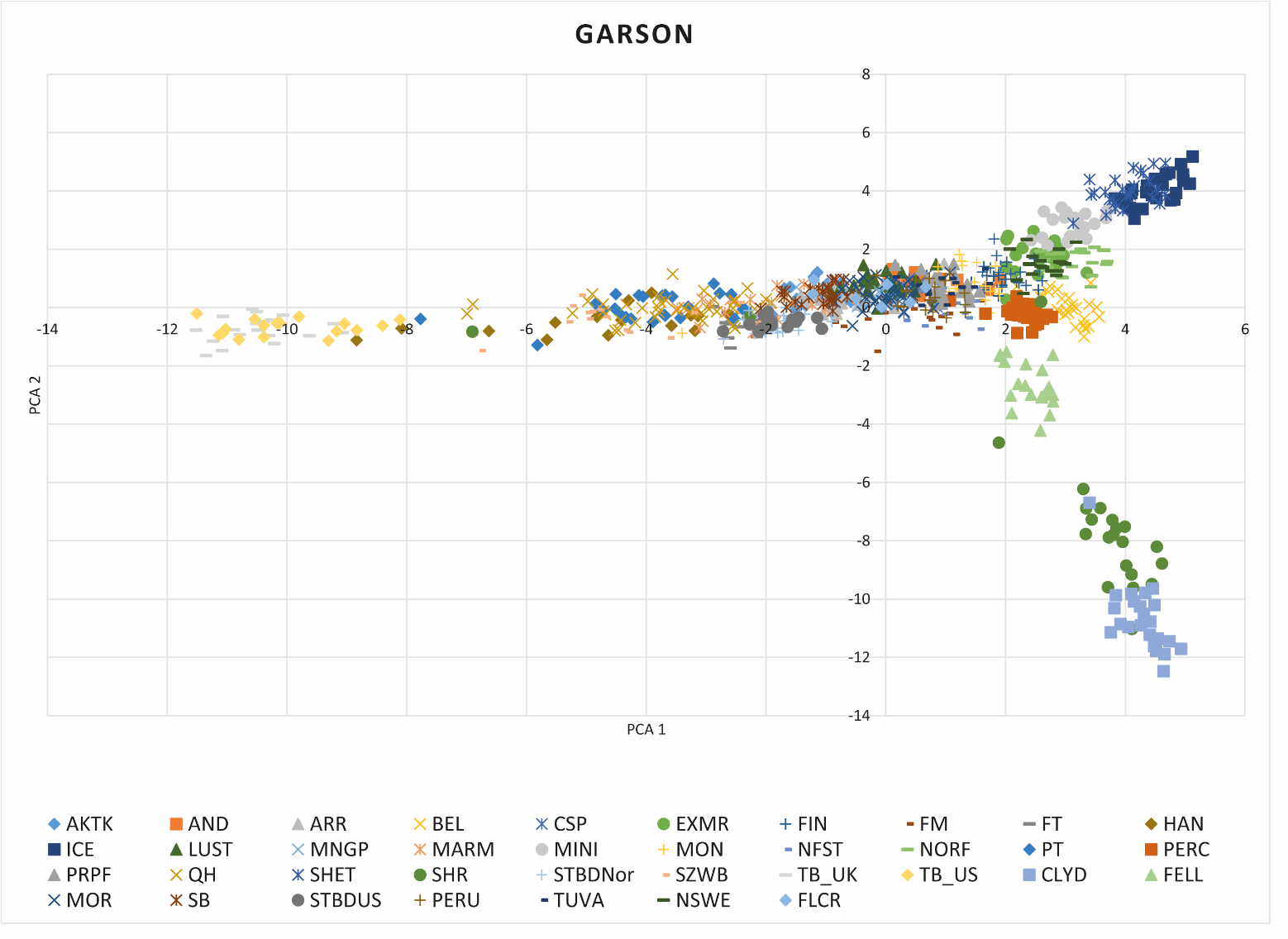


Figure S2 Animals were clustered based on principal components analysis (PCA) using 4940 SNP markers (Garson). PC1 and PC2 are shown on the X-axis the Y-axis respectively. The horse breeds demarcated using unique and different symbols and colors.


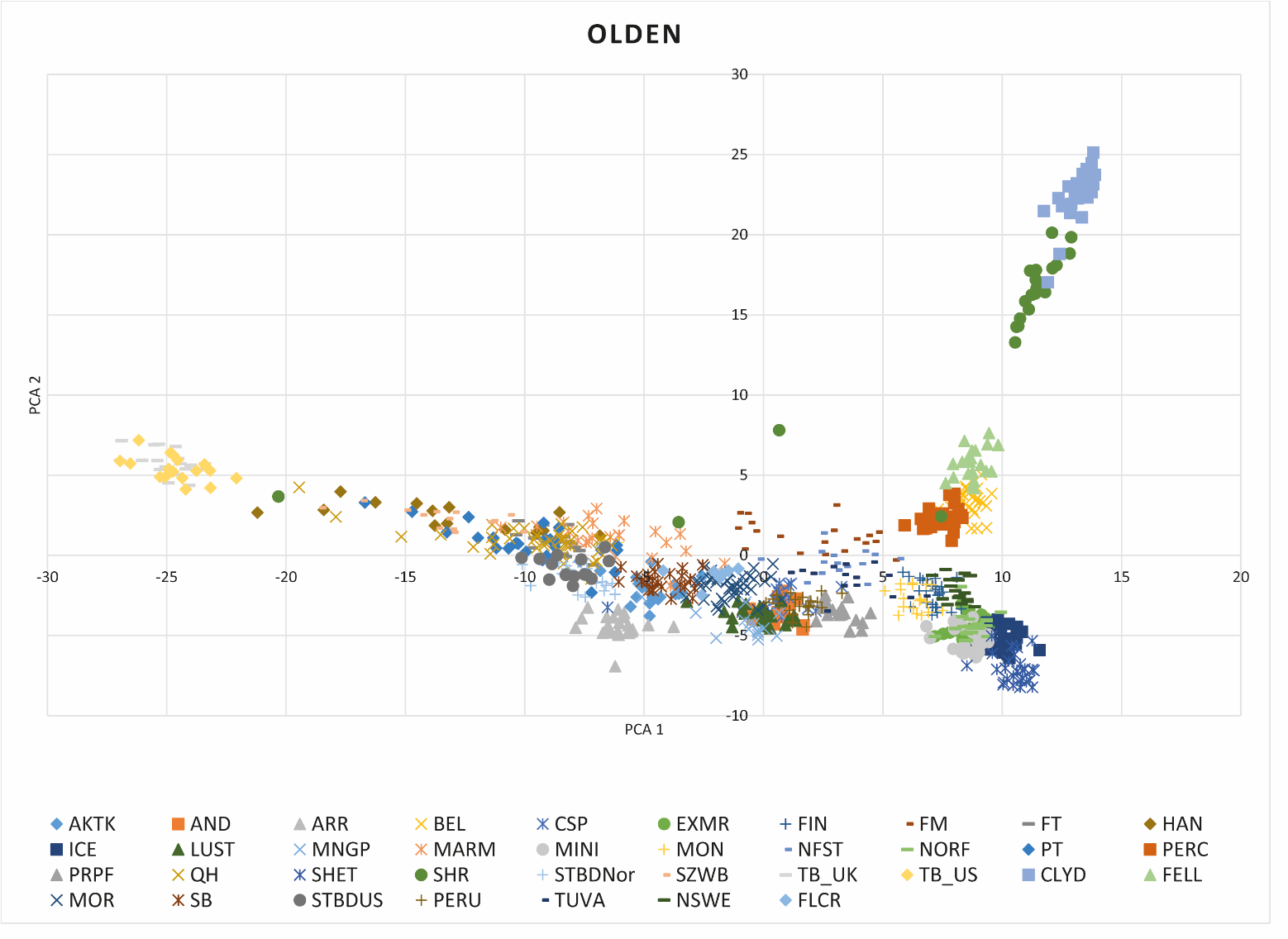


Figure S3 Animals were clustered based on principal components analysis (PCA) using 7999 SNP markers (Olden). PC1 and PC2 are shown on the X-axis the Y-axis respectively. The horse breeds demarcated using unique and different symbols and colors.

**Correct assignment of the individuals**

Table S1 Correct assignment of an individual by three Methods.

| SNP-No |  | LLR > 1 | | |  | LLR > 2 | | |  | LLR > 3 | | |  | LLR > 4 | | |
| --- | --- | --- | --- | --- | --- | --- | --- | --- | --- | --- | --- | --- | --- | --- | --- | --- |
|  |  | **DNN** | **Olden** | **Garson** |  | **DNN** | **Olden** | **Garson** |  | **DNN** | **Olden** | **Garson** |  | **DNN** | **Olden** | **Garson** |
| **50** |  | 353 (44.4) | 50 (6.29) | 100 (12.58) |  | 191 (24.03) | 38 (4.78) | 64 (8.05) |  | 93 (11.7) | 31 (3.9) | 39 (4.91) |  | 35 (4.4) | 17 (2.14) | 27 (3.4) |
| **75** |  | 524 (65.91) | 130 (16.35) | 177 (22.26) |  | 377 (47.42) | 92 (11.57) | 119 (14.97) |  | 272 (34.21) | 73 (9.18) | 93 (11.7) |  | 171 (21.51) | 44 (5.53) | 74 (9.31) |
| **100** |  | 624 (78.49) | 274 (34.47) | 256 (32.2) |  | 510 (64.15) | 190 (23.9) | 190 (23.9) |  | 395 (49.69) | 151 (18.99) | 152 (19.12) |  | 298 (37.48) | 110 (13.84) | 123 (15.47) |
| **125** |  | 681 (85.66) | 451 (56.73) | 358 (45.03) |  | 613 (77.11) | 319 (40.13) | 269 (33.84) |  | 518 (65.16) | 256 (32.2) | 219 (27.55) |  | 433 (54.47) | 187 (23.52) | 181 (22.77) |
| **150** |  | 722 (90.82) | 550 (69.18) | 483 (60.75) |  | 668 (84.03) | 434 (54.59) | 381 (47.92) |  | 600 (75.47) | 354 (44.53) | 311 (39.12) |  | 531 (66.79) | 282 (35.47) | 258 (32.45) |
| **200** |  | 755 (94.97) | 705 (88.68) | 629 (79.12) |  | 740 (93.08) | 641 (80.63) | 546 (68.68) |  | 706 (88.81) | 568 (71.45) | 483 (60.75) |  | 668 (84.03) | 492 (61.89) | 426 (53.58) |
| **250** |  | 760 (95.6) | 761(95.72) | 698 (87.8) |  | 751 (94.47) | 735 (92.45) | 657 (82.64) |  | 727 (91.45) | 699 (87.92) | 604 (75.97) |  | 701 (88.18) | 663 (83.4) | 547 (68.81) |
| **300** |  | 774 (97.36) | 774 (97.36) | 759 (95.47) |  | 762 (95.85) | 766 (96.35) | 727 (91.45) |  | 745 (93.71) | 752 (94.59) | 697 (87.67) |  | 732 (92.08) | 731 (91.95) | 653 (82.14) |
| **350** |  | 781 (98.24) | 776 (97.61) | 776 (97.61) |  | 771 (96.98) | 770 (96.86) | 760 (95.6) |  | 759 (95.47) | 761 (95.72) | 742 (93.33) |  | 745 (93.71) | 746 (93.84) | 712 (89.56) |
| Number of individuals assigned correctly (percentage) | | | | | | | | | | | | | | | | |

**PCA plot of each method – Training data**


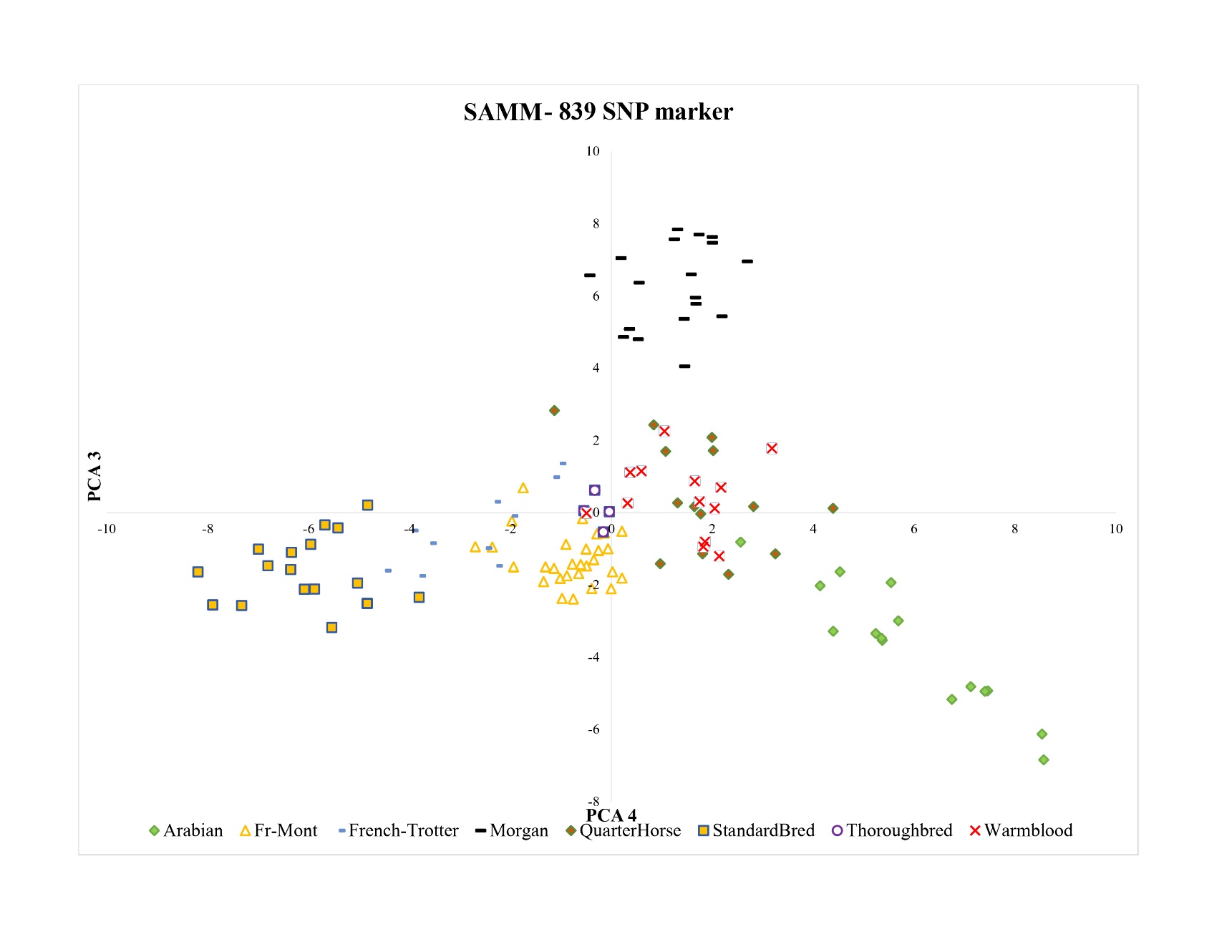


Figure S4 Animals were clustered based on principal components analysis (PCA) using 839 SNP markers (DNN-Validation data). PC4 and PC3 are shown on the X-axis the Y-axis respectively. The horse breeds demarcated using unique and different symbols and colors.


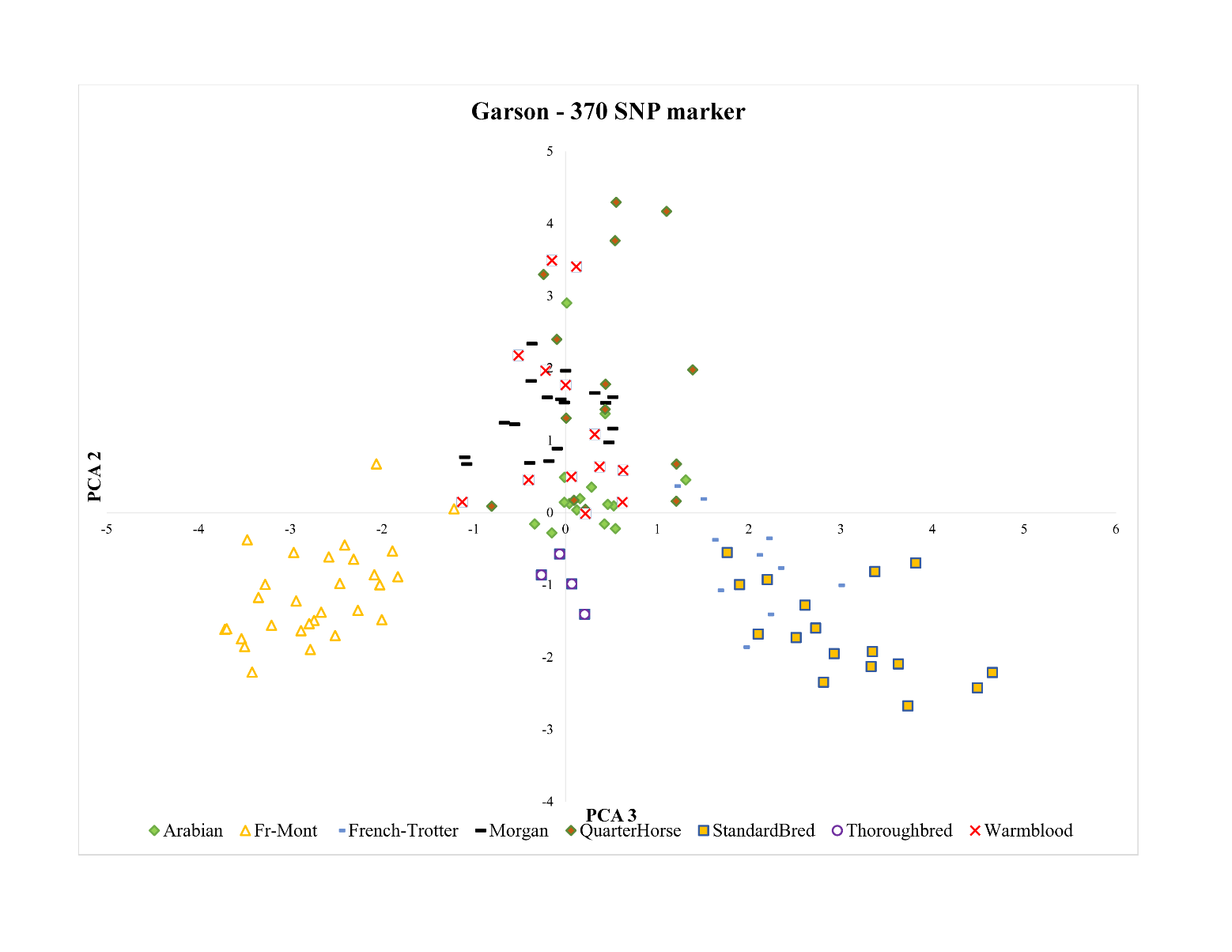


Figure S5 Animals were clustered based on principal components analysis (PCA) using 370 SNP markers (Garson-Validation data). PC3 and PC2 are shown on the X-axis the Y-axis respectively. The horse breeds demarcated using unique and different symbols and colors.


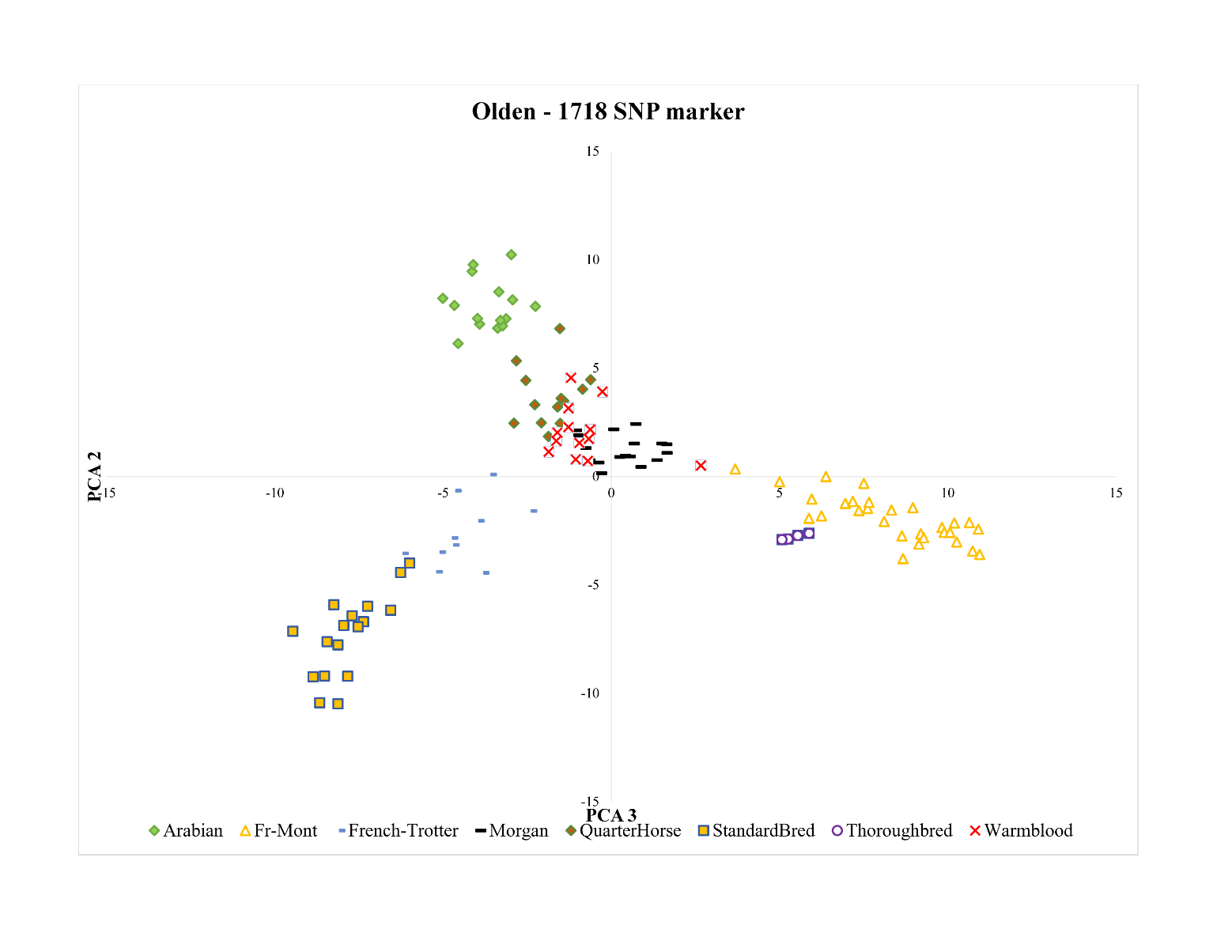


Figure S6 Animals were clustered based on principal components analysis (PCA) using 1718 SNP markers (Olden-Validation data). PC3 and PC2 are shown on the X-axis the Y-axis respectively. The horse breeds demarcated using unique and different symbols and colors.

**Neighbor-Joining tree of allele-sharing distances**


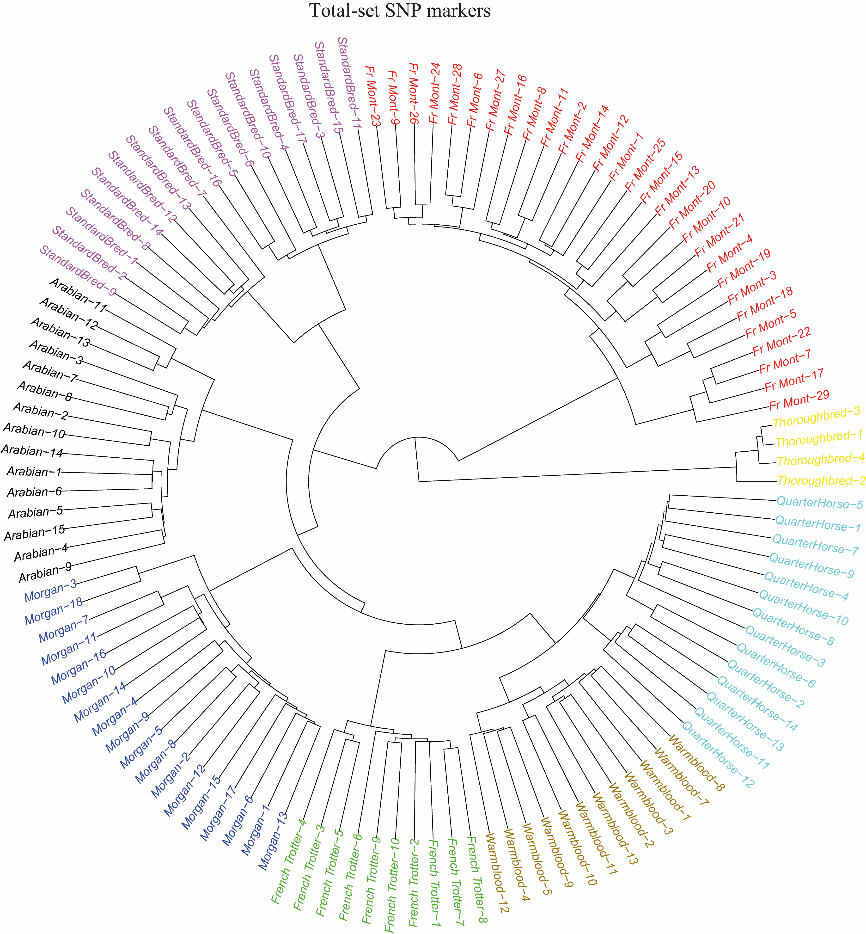


Figure S7 Neighbor-Joining tree plot by allele-sharing distances (32419 SNP Markers).


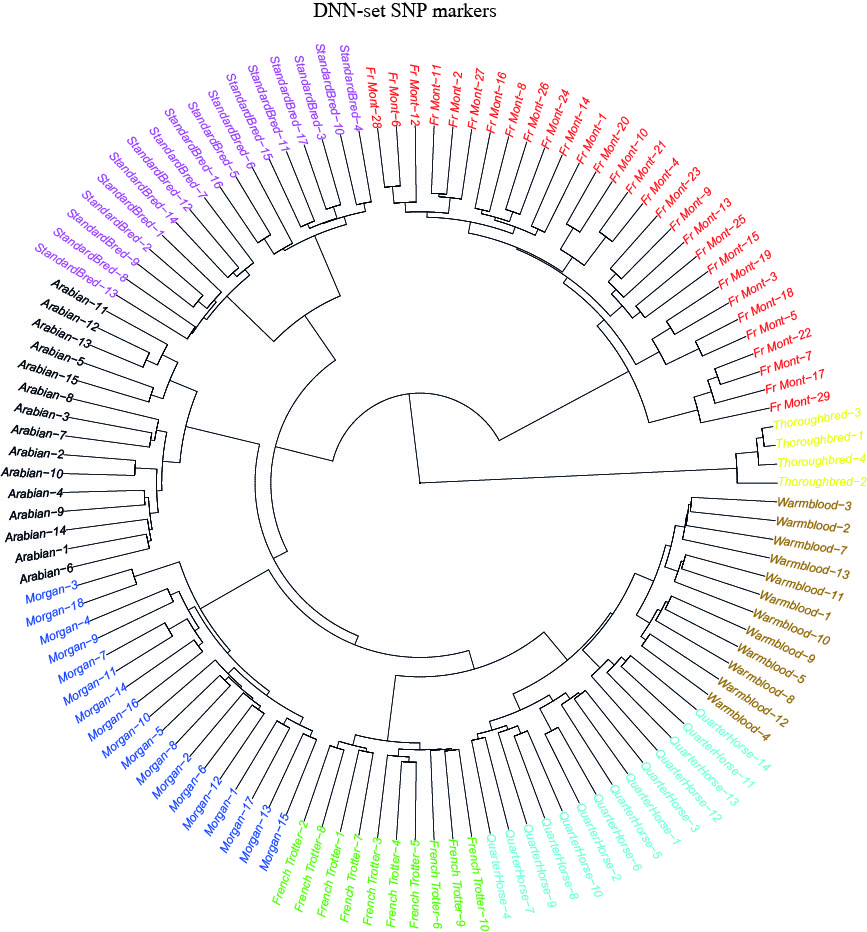


Figure S8 Neighbor-Joining tree plot by allele-sharing distances (Selected SNP Markers by DNN method).


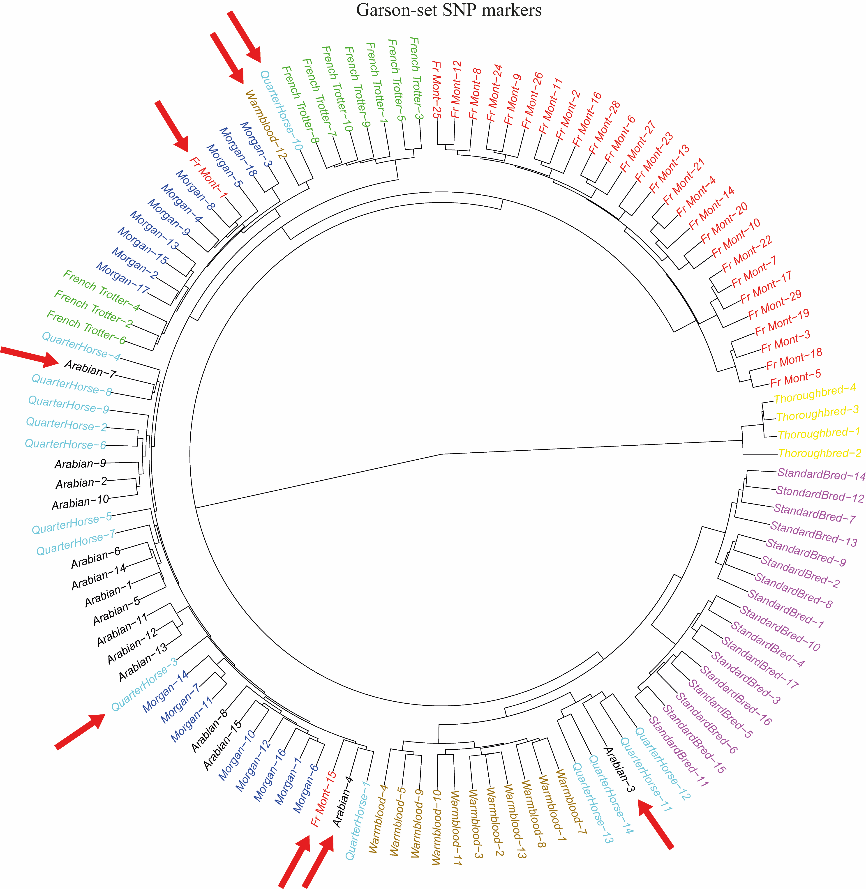


Figure S9 Neighbor-Joining tree plot by allele-sharing distances (Selected SNP Markers by Garson method).


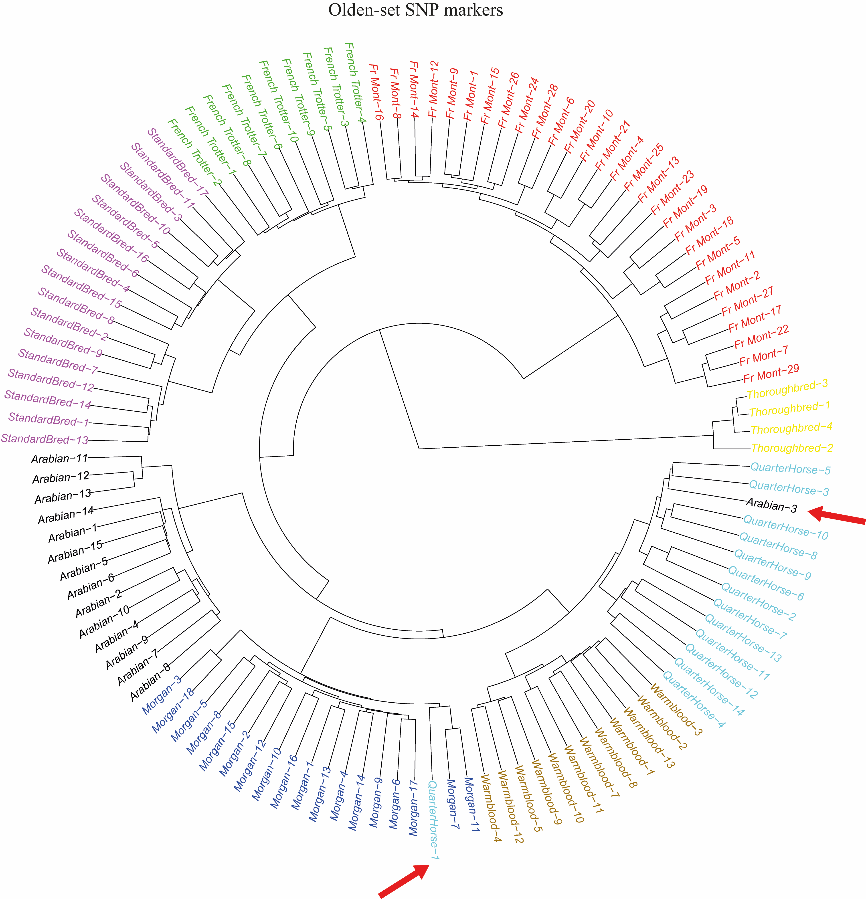


Figure S10 Neighbor-Joining tree plot by allele-sharing distances (Selected SNP Markers by Olden method).

**Supplementary Information Captions**

**Figure S1** Animals were clustered based on principal components analysis (PCA) using 4270 SNP markers (DNN). PC1 and PC2 are shown on the X-axis and the Y-axis respectively. The horse breeds are demarcated using unique and different symbols and colors.

**Figure S2** Animals were clustered based on principal components analysis (PCA) using 4940 SNP markers (Garson). PC1 and PC2 are shown on the X-axis and the Y-axis respectively. The horse breeds are demarcated using unique and different symbols and colors.

**Figure S3** Animals were clustered based on principal components analysis (PCA) using 7999 SNP markers (Olden). PC1 and PC2 are shown on the X-axis and the Y-axis respectively. The horse breeds are demarcated using unique and different symbols and colors.

**Figure S4** Animals were clustered based on principal components analysis (PCA) using 839 SNP markers (DNN-Validation data). PC4 and PC3 are shown on the X-axis and the Y-axis respectively. The horse breeds are demarcated using unique and different symbols and colors.

**Figure S5** Animals were clustered based on principal components analysis (PCA) using 370 SNP markers (Garson-Validation data). PC3 and PC2 are shown on the X-axis and the Y-axis respectively. The horse breeds are demarcated using unique and different symbols and colors.

**Figure S6** Animals were clustered based on principal components analysis (PCA) using 1718 SNP markers (Olden-Validation data). PC3 and PC2 are shown on the X-axis and the Y-axis respectively. The horse breeds are demarcated using unique and different symbols and colors.

**Figure S7** Neighbor-Joining tree plot by allele-sharing distances (32419 SNP Markers)

**Figure S8** Neighbor-Joining tree plot by allele-sharing distances (Selected SNP Markers by Deep Neural Network)

**Figure S9** Neighbor-Joining tree plot by allele-sharing distances (Selected SNP Markers by Garson method)

**Figure S10** Neighbor-Joining tree plot by allele-sharing distances (Selected SNP Markers by Olden method)
